# Large-scale profiling of signalling pathways reveals an asthma specific signature in bronchial smooth muscle cells

**DOI:** 10.18632/oncotarget.7209

**Published:** 2016-02-05

**Authors:** Elena Alexandrova, Giovanni Nassa, Giacomo Corleone, Anton Buzdin, Alexander M. Aliper, Nadezhda Terekhanova, Denis Shepelin, Alexander Zhavoronkov, Michael Tamm, Luciano Milanesi, Nicola Miglino, Alessandro Weisz, Pieter Borger

**Affiliations:** ^1^ Laboratory of Molecular Medicine and Genomics, Department of Medicine and Surgery, University of Salerno, Baronissi (SA), Italy; ^2^ Genomix4Life Srl, Campus of Medicine, University of Salerno, Baronissi (SA), Italy; ^3^ Laboratory of Bioinformatics, D. Rogachyov Federal Research Center of Pediatric Hematology, Oncology and Immunology, Moscow, Russia; ^4^ Pathway Pharmaceuticals, Wan Chai, Hong Kong, Hong Kong SAR; ^5^ Group for Genomic Regulation of Cell Signalling Systems, Shemyakin-Ovchinnikov Institute of Bioorganic Chemistry, Moscow, Russia; ^6^ Insilico Medicine, Inc, ETC, Johns Hopkins University, Baltimore, MD, USA; ^7^ Department of Biomedicine, University Hospital Basel, Basel, Switzerland; ^8^ Institute of Biomedical Technologies, National Research Council, Segregate, (MI), Italy; ^9^ Molecular Pathology and Medical Genomics Unit, ‘SS. Giovanni di Dio e Ruggi d'Aragona - Schola Medica Salernitana’ University Hospital, Salerno, (SA), Italy

**Keywords:** asthma, smooth muscle cells, signalling pathways, CAGE

## Abstract

**Background:**

Bronchial smooth muscle (BSM) cells from asthmatic patients maintain *in vitro* a distinct hyper-reactive (“primed”) phenotype, characterized by increased release of pro-inflammatory factors and mediators, as well as hyperplasia and/or hypertrophy. This “primed” phenotype helps to understand pathogenesis of asthma, as changes in BSM function are essential for manifestation of allergic and inflammatory responses and airway wall remodelling.

**Objective:**

To identify signalling pathways in cultured primary BSMs of asthma patients and non-asthmatic subjects by genome wide profiling of differentially expressed mRNAs and activated intracellular signalling pathways (ISPs).

**Methods:**

Transcriptome profiling by cap-analysis-of-gene-expression (CAGE), which permits selection of preferentially capped mRNAs most likely to be translated into proteins, was performed in human BSM cells from asthmatic (n=8) and non-asthmatic (n=6) subjects and OncoFinder tool were then exploited for identification of ISP deregulations.

**Results:**

CAGE revealed >600 RNAs differentially expressed in asthma *vs* control cells (p≤0.005), with asthma samples showing a high degree of similarity among them. Comprehensive ISP activation analysis revealed that among 269 pathways analysed, 145 (p<0.05) or 103 (p<0.01) are differentially active in asthma, with profiles that clearly characterize BSM cells of asthmatic individuals. Notably, we identified 7 clusters of coherently acting pathways functionally related to the disease, with ISPs down-regulated in asthma mostly targeting cell death-promoting pathways and up-regulated ones affecting cell growth and proliferation, inflammatory response, control of smooth muscle contraction and hypoxia-related signalization.

**Conclusions:**

These first-time results can now be exploited toward development of novel therapeutic strategies targeting ISP signatures linked to asthma pathophysiology.

## INTRODUCTION

A major hallmark of the asthmatic airway wall is the increased bulk of airway smooth muscle (BSM) cells. *In vitro*, it has been shown that BSM cells of asthmatic patients have a distinct hyper-reactive (“primed”) phenotype, which is characterized by an increased release of pro-inflammatory factors and mediators, as well as hyperplasia and/or hypertrophy [1-3]. The “primed” phenotype of BSM cells might be pivotal to understanding the pathology of asthma, as changes in BSM function are essential for the manifestation of allergic and inflammatory responses and airway wall remodelling [4, 5].

Despite increasing evidence pointing to aberrant signalling mechanisms within BSM cells of asthmatic subject [6-8], only few studies have identified downstream signalling cascades that serve to control contractility and relaxation of BSM cells [9], BSM cell proliferation and apoptosis [7, 10, 11], as well as BSM cell-released extracellular components [12] and mediators that regulate immunity and angiogenesis [13]. The diversity of functions of BSM cells may thus modulate bronchomotor tone, airway hyper-responsiveness, as well as inflammatory and remodelling responses [14]. Together, this broad range of observations provide a rationale for regarding BSM cells as a major effector cells of the asthmatic lung and disturbed signalling in these cells as a major research target.

We recently developed a new bio-informatics approach called OncoFinder, which enables quantitative measurement of intracellular signalling pathway (ISP) activation basing on gene expression data [15-17]. OncoFinder performs a quantitative measurement of the signalling pathway activation termed “pathway activation strength” (PAS) [15,18]. PAS measures the cumulative value of perturbations in a signalling pathway and serves as an indicator of pathological changes in the intracellular signalling machinery [19]. The PAS value itself serves as a new type of biomarker that can distinguish between the pathway activation profiles in different tissue types [20].

We have hypothesized and provided evidence that the “primed” phenotype of asthmatic BSM cells is reflected in the signalling pathway signature of the cells. Using small RNA profiling we recently identified deregulated phosphatase and tensin homolog (PTEN)/phosphoinositide 3-kinase (PI3K)/Akt pathways in BSM cells of asthmatic patients as a candidate for a primed phenotype [21].

Here, we performed large-scale profiling of signalling pathway activation signatures in BSM cells of asthmatic and non-asthmatic subjects. Using the cap analysis of gene expression (CAGE) technique, which exclusively identifies 5′-capped mRNAs, complementary to RNA-seq [22], and therefore more accurately quantifies the proportion of mRNAs being translated into proteins, we compared the transcriptomes of primary BSM cells of asthmatic and non-asthmatic subjects. Our data show that unsupervised hierarchical clustering of CAGE tag cluster expression profiles as well as differentially activated ISPs, including the PTEN/AKT signalling system, clearly distinguished asthmatic from non-asthmatic samples.

## RESULTS

### Subjects characteristics

Patients with mild to moderate asthma (n = 8; 3 females/5 males, age 23-77 years) had reversible airway obstruction documented in the past with median FEV1 of 75.3% of the predicted value (ranging from 35.2% to 86.7%; Table 1). Cross-sections of human airway wall tissue demonstrated a marked increase of the bulk of smooth muscle bundles in asthmatic relative to non-asthmatics (data not shown).

**Table 1:** Characteristics of asthma patients (All Caucasian)

Sample N	Diagnosis	Age	Gender	FEV1^1^(%)	Treatment
**638**	Asthma	34	Male	86.7	None
**662**	Asthma	36	Male	76.2	Symbicort^2^ (=ICS^3^+LABA^4^)
**667**	Asthma	51	Female	58.2	None
**669**	Asthma	23	Male	75.3	Symbicort (=ICS+LABA)
**670**	Asthma	37	Female	ND	None
**695**	Asthma	56	Male	87.4	None
**738**	Asthma	77	Female	41.2	None
**739**	Asthma	64	Male	35.2	Symbicort (=ICS+LABA)

1 FEV1 – Forced Expiratory Volume in 1 second

2 Symbicort - a combination of anti-inflammatory and bronchodilator medicines.

3 ICS – Inhaled Corticosteroids

4 LABA – Long-Acting Beta Agonists

### Cap analysis of gene expression (CAGE) of asthmatic and normal BSM cells

In order to isolate 5′-capped RNA, we constructed CAGE libraries using total RNA of BSM cells from asthmatic and controls subjects [23]. On average, 24 millions of reads were obtained for each library (Supplementary Table S1). Since a key step of CAGE library preparation is the enzymatically cleavage of 5′-adapter-ligated double-stranded cDNAs with Type III restriction endonuclease EcoP15I, which cleaves 27nt apart from its binding site, placed at the 3′end of adapter sequence, CAGE libraries should have basically the same read length after adapter trimming. To evaluate if this was true also for the libraries made for this study, we analysed distribution of sequenced read lengths after adapter trimming, confirming that the majority of the sequenced reads did have the expected length of 27 base pairs (Supplementary Figure S1A). Confirming the high quality of obtained reads is also the fact that average sequence quality scores (PHRED scores) was >30 (Supplementary Figure S1B).

### Transcriptional landscape of asthma BSM cells

**Figure 1:**
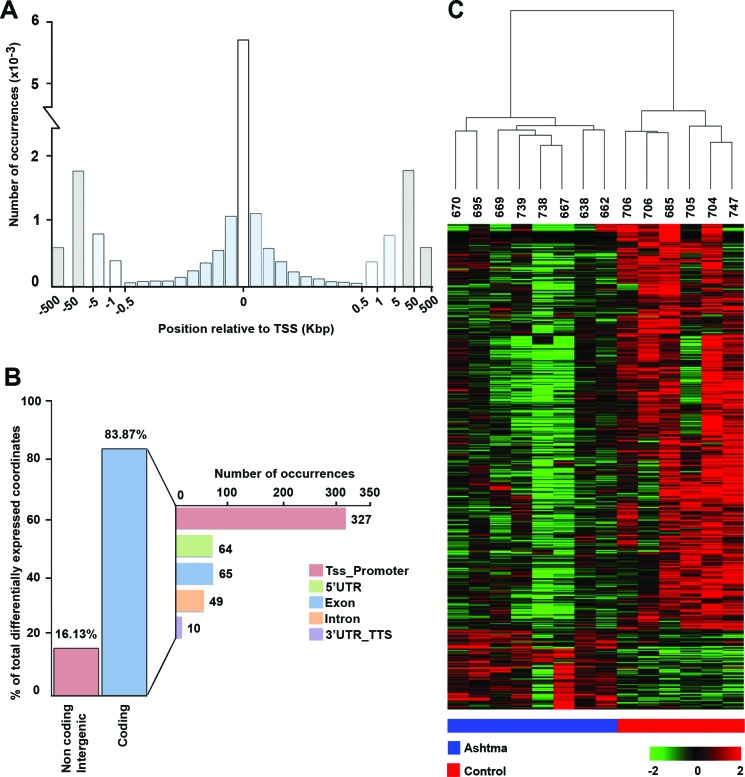
BSM transcription start sites recapitulate known transcription initiation and reveal gene expression modulation in asthma **A.** Distribution of distances between CAGE TSSs and the closest 5′ end of a transcript annotated in Ensembl database. **B.** Histogram summarizing the proportion of differentially expressed CAGE tag clusters (CTCs) aligned to non-coding transcripts or intergenic regions and to protein-coding transcripts. **C.** Unsupervised hierarchical clustering of differentially expressed CTCs.

The analysis of distribution of distances between CAGE tags and the closest 5′ end of annotated transcripts is reported in Figure 1A. The histogram in the figure shows number of tags mapped to the first 50 bases upstream or downstream of known TSSs (column “0”), next columns to the left and right of “0” show number of tags that map between 51 and 100 bases downstream from TSS in both directions (being genes encoded in either DNA strand) and so on, up to 500 bp (0.5 kbp). The data shown represent number of tags upstream and downstream to TSSs, mapped at the following distances: 501 to 1.000 bp (1 kbp), 1.001 to 5.000 bp (5 kbp), 5.001 to 50.000 bp (50 kbp) or 50.001 to 500.000 bp (500 kbp) away from TSSs. The analysis led to mapping of the CAGE tags within or proximal to >5,600 documented transcription start sites (TSSs). As the distance from TSSs increased, a significant decrease in mapped CAGE tags can be observed (Figure 1A), as expected for this type of analysis. Differential expression analysis revealed a total of 614 differentially expressed CAGE tag clusters (CTCs; p-value ≤0.005), including 515 down-regulated and 99 are up-regulated in BSM cells from asthma patients (see Supplementary Table S2, that includes also FDR values). Analysis of transcripts associated with differentially expressed CTCs showed that 84% of them represent protein coding mRNAs and 16% corresponding to noncoding, or intergenic, transcripts (Figure 1B). For tags mapped to coding mRNAs, CTCs associated to promoters (defined as the region spanning from 1 to 500 bp upstream of the gene) of annotated transcripts, or to the 5′ UTR, exons, introns and 3′UTRs were identified. Out of 515 CTCs aligning to mRNAs, 327, 64, 65, 49 and 10 derive from TSS-proximal regions, 5′ UTRs, exons, introns or 3′ UTR/transcriptional termination sites (3′UTR_TTS in the figure), respectively (Figure 1B). Unsupervised hierarchical clustering analysis based on tag clusters expression was performed with the Ward's agglomeration method, operated on Kendall correlation distance measures. It demonstrated that asthma and control samples cluster in two widely separate groups, with samples showing in each case a very similar expression profile among them. Results of this analysis are summarized in Figure 1C, where each row corresponds to one transcript and each column corresponds to one sample. Red and blue lines below the heatmap mark asthma and control samples, respectively.

### Building intracellular signalling pathway activation profiles

**Figure 2:**
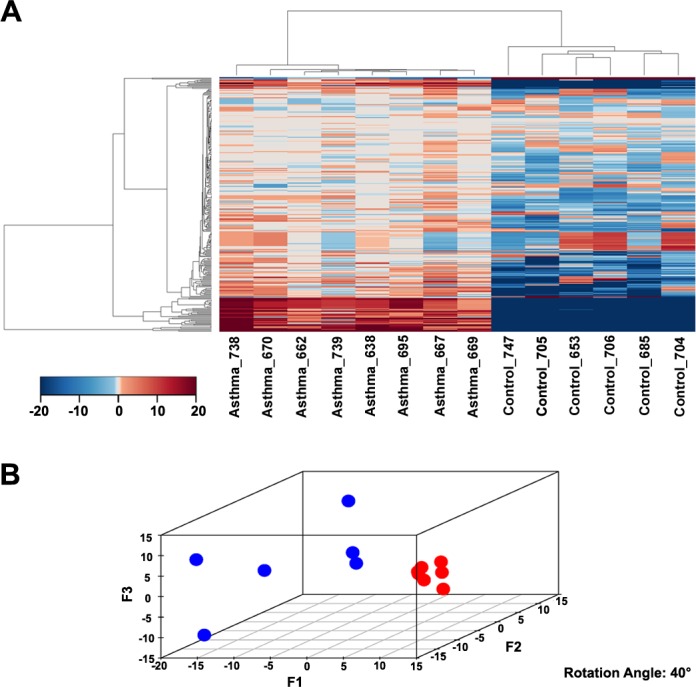
Modulation of signalling pathway activity in asthma **A.** Hierarchical clustering heatmap based on the analysis of 269 intracellular signalling pathway activation profiles. Color key represents PAS score for a given pathway in a given sample. **B.** Results of the principal component analysis. Six non-asthmatic and eight asthmatic BSM samples were analysed according to their PAS score signatures calculated for 269 intracellular signalling pathways. Blue dots denote normal samples, red – asthmatic samples.

The normalized gene expression levels of six non-asthmatic and the eight asthmatic subjects are shown in Supplementary Table S3. These data were processed using the OncoFinder algorithm to establish pathway activation strength (PAS) profiles. We analysed the activation status of 269 intracellular signalling pathways (described in Supplementary Table S4). The PAS data, together with corresponding p-values and FDRs, are shown in Supplementary Table S5. Basing on these results, we built hierarchical clustering heatmap with Euclidean distance and average linkage for all the samples investigated (Figure 2A). It is clear that asthmatic and non-asthmatic BSM samples display two distinct and isolated clusters. This indicates that asthma is associated with multiple common changes in signalling pathway activation; changes that are shared by all (or the most) of the asthma samples. This observation was confirmed by the Principal Component Analyses, where asthma samples formed an unmistakably defined group in the plot (Figure 2B).

### Functional significance of pathway activation analysis

**Figure 3:**
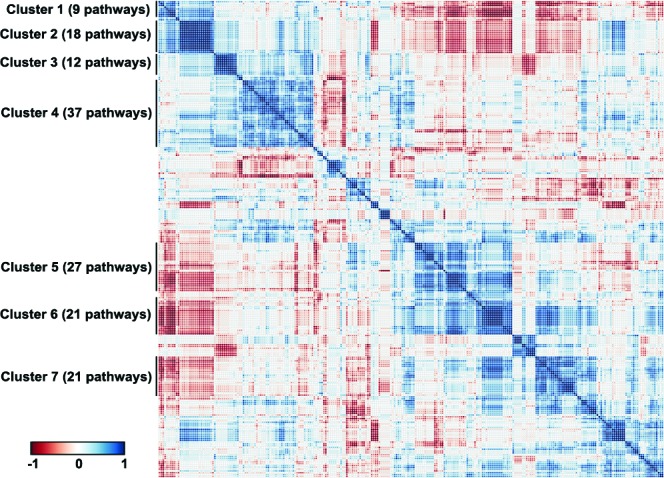
Signalling pathways correlation plot Correlation plot of the activities of 269 intracellular signalling pathways among the fourteen investigated normal and asthmatic BSM tissue samples. Seven clusters of commonly regulated pathways are indicated on the figure. Color key represents correlation coefficients between the pathways ranging from -1 to 1.

**Figure 4:**
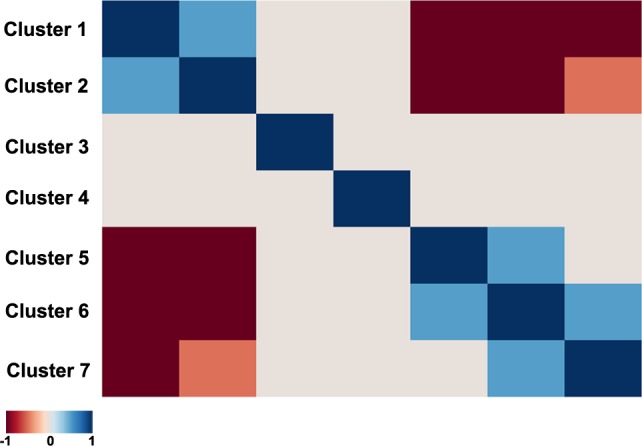
Heatmap of the functional interactions between the signalling pathway clusters Clusters are given from 1 to 7 (left to right and top down). Correlation coefficients between the Clusters activation profiles vary in the interval from -1 till 1, corresponding to variation from dark red till dark blue on the heatmap, according to the defined color key.

For 145 (p<0.05) and 103 (p<0.01) pathways, we detected their differential activation between asthmatic and non-asthmatic BSM cells (Supplementary Table S5). The major part of the differentially activated signalling pathways (136/145; 94%) were up-regulated in asthma, whereas only 9/145 (6%) were down-regulated. Down-regulated pathways in asthma mainly represented cell death-promoting pathways, whereas the up-regulated ones were predominantly involved in cell growth and proliferation, inflammatory response and some specific reactions, including smooth muscle contraction and hypoxia-associated signalling. The individual data points of all 269 investigated ISPs are presented in Supplementary Table S4. As demonstrated in Figure 3, the correlation plot of the activities of 269 intracellular signalling pathways identified 7 clusters of co-regulated pathways among the 14 investigated samples. The identity of signalling pathway that form the 7 distinct clusters are offered in Supplementary Table S6. Finally, we constructed a heatmap and determined correlation coefficients between the clusters to identify functional interactions. As shown in Figure 4, the activities of clusters 1 and 2 are positively correlated with each other, but negatively with the activities of clusters 5, 6 and 7. Likewise, the activities of clusters 5, 6 and 7 are positively correlated to each other, but negatively correlated to clusters 1 and 2.

## DISCUSSION

This study is the first to systematically profile differentially activated ISPs in cultured primary human BSM cells from asthmatic and non-asthmatic subjects using the CAGE technique, highlighting asthma-specific co-regulatory patterns. The 5′-capping of transcribed RNAs is a fundamental prerequisite for RNAs to be translated into proteins, as translation commences with binding of eIF4F protein complex to mRNA 5′-end cap (m^7^GpppN, where N is any nucleotide and m is a methyl group), followed by mRNA 5′UTR region unwinding that further facilitates binding to the 40S ribosomal subunit [24]. Thus, analysis of 5′-capped RNAs, rather than of the entire RNA pool, has the advantage that it focuses only on translation-ready RNAs, excluding non-coding RNAs and pseudo-genes, which are often difficult to distinguish from protein-coding gene transcripts. The exclusive identification of 5′-capped mRNAs provided us expression datasets better matching protein expression levels in the BSM cells.

The distribution of distances between CAGE tags and the closest 5′ end of annotated transcripts (Figure 1A) demonstrated that the vast majority of CAGE tags for all expressed transcripts mapped within 50 bases from the known TSSs of corresponding genes, which is in agreement with typical tag distribution profile generally obtained for CAGE libraries [25]. The proportion of CAGE tags mapped at the distance of 50 to 500 kbp to the closest known TSSs may represent unannotated genes.

Differential expression analysis showed that, with 614 differentially expressed transcripts (p≤0.005), the expression profiles of the transcripts are distinctly different between asthma and control BSM cells. Of those, 515 were down- and 99 were up-regulated in asthma (Figure 1C and listed in Supplementary Table S2 which also shows the FDR values for each gene). Analysis of the nature of transcripts associated with differentially expressed CTCs showed that 16% represented non-coding or intergenic RNAs, whereas 84% coded for protein (Figure 1B). Further, unsupervised hierarchical clustering analysis revealed that the asthmatic patients can be clearly distinguished from control subjects based on CTC expression patterns (Figure 1C). Noteworthy, the dendrogram presented above the heatmap indicates that the distance between samples of the same group (asthma or control) is much smaller than the distance between samples of different groups, hence confirming that BSM cells of asthmatic and non-asthmatic subjects really form two distinct populations. Of the 269 pathways we investigated, a significant fraction was differentially activated in asthmatic BSM cells, the majority being more active. As observed for the CMC signatures, the pathway activation data (heatmap, Figure 2A) also clearly distinguished between BSM cells of asthmatic and non-asthmatic subjects. In accord with our previous studies analysing small noncoding RNA expression patterns [21], the PTEN/AKT signalling system was again amongst the most affected ISPs.

The down-regulated pathways in asthma predominantly represented cell death-promoting pathways, whereas up-regulated pathways generally induce or enhance cell proliferation and growth, vascularization, inflammatory response and muscle-tissue specific responses, such as contractility. Moreover, we show here, for the first time, that the activity of 135 signalling pathways forms seven clearly distinguishable clusters, each characterized by concordant activation signatures of the underlying pathways. Only a tiny fraction of this clustering can be explained by similarities in gene content between the pathways. This suggests a true functional coordination between the cluster members. Further analysis of these functional clusters in view of building “super-pathways” - accumulating concordant functionally linked signalling pathways - goes beyond the aim of this study (molecular interactions in asthma), but our study showed that such is possible and may be potentially of great biological significance.

The hierarchical clustering and principal component analyses demonstrated that the asthmatic BSM samples form more tight clusters relative to the non-asthmatic controls (Figure 2B). This means that asthma is characterized by a more uniform signalling pathway activation signature than for the non-asthmatic condition samples. This is an intriguing and unexpected finding, because asthma is generally regarded as a heterogeneous pulmonary disorder involving many cell types [26], numerous genetic associations [27], and the disease can manifest itself as several different phenotypes [28]. Our data now indicate that, despite this heterogeneity, disturbed molecular signalling mechanisms of BSM cells are highly similar among individual asthma patients: Asthmatic BSM cells are more similar to each other than control cells are to each other, indicating similar molecular changes causing an asthma phenotype. The uniformity of signalling pathway activation may reflect the common manifestation of airway constriction and BSM hyperplasia present in all asthma patients. Importantly, this observation envisages accomplishment on finding a set of universal anti-asthmatic molecular targets in the future.

Up-regulated pathways were mostly responsible for promotion of cell growth (e.g., mTOR, IGF1R, Growth Hormone pathways), survival (e.g., Akt pathway, cellular anti-apoptotic mechanisms) and proliferation (e.g., Ras, MAPK, Spindle assembly and chromosome separation pathways), enhanced vascularization (VEGF pathway), for inducing inflammatory response (IL2, IL6, IL10, CD40, Interferon pathways) and other processes like increased muscle contractility (Muscle contraction-specific branch of cAMP pathway). Asthma samples were also enriched in the activities linked with RNA transcription (e.g. Transcription of RNA pathway and RNA polymerase II complex) and protein translation (e.g. branch of mTOR pathway positively regulating translation and positive regulation of translation initiation via eIF4F translation factor pathway). We also detected increased proteasome-mediated protein degradation (ubiquitin-proteasome pathway) and enhanced autophagy (PPAR pathway) in asthma BSMs. Although PPARgamma has been shown to be increased in BSM asthma after mitogenic stimulation, it was not related to the increased proliferation observed in asthmatic BSM [28]. In contrast, pathways preventing cell growth (SMAD pathway branch leading to degradation of cell surface receptors of growth factors), promoting cell cycle arrest (PTEN pathway), cell death (Caspases cascade and Mitochondrial apoptosis pathway), were significantly down-regulated in asthmatic samples (Supplementary Table S5; description of pathways is given in Supplementary Table S4). Together, these observations provide a rational for the observation that *in vitro* cultured BSM cells of asthma patients grow faster than their non-asthmatic counterparts [3, 7, 29] and may thus explain the *in vivo* observation of the increased bulk of BSM cells present in the airway wall.

Among the affected signalling processes, some were single pathways (e.g., Notch and EGFR pathways enhanced in asthma), whereas others were represented by multiple terminal branches of the same pathway (e.g. Glucocorticoid hormone pathway and its four terminal branches upregulated in asthma; Integrin linked kinase (ILK) pathway and its eleven terminal branches upregulated in asthma; Supplementary Table S5). Importantly, many of the pathways presented in Supplementary Table S5 do not come as a surprise, since they have previously been reported in association with asthma. Among them are those that are associated with β2-adrenoceptor agonists (e. g. GPCR-, cAMP-, CREB-, RAS- and p38-pathway) and glucocorticosteroids (e. g. androgen hormone-, IP3-, RAS-, and mTOR-pathway), as well as the more general cell activation cascades induced by growth hormones, cytokines and chemokines (e. g. chemokine-, JNK-, PAK-, p38-, ERK-and Jak/Stat-pathway). The androgen hormone pathway is of particular interest, since glucocorticosteroids (GS) are known to reduce airway contraction through altering calcium mobilization or Na+/K+ ATPase potentiation. GS also alter the formation of IP3 and cAMP levels through down-regulation and/or uncoupling of G-protein coupled receptors, thus exerting profound effects on the secretory and proliferating activity of BSM cells (reviewed in [30]). Indeed, glucocorticoids are very effective in reducing the proliferation of BSM cells [31]. Likewise, the anti-mitogenic effects induced by GCPR-cAMP system involve numerous pathways, including inhibition of ERK1/2 and phosphoinositide 3′-kinase (PI3K), via PKA activation and Epac in BSM cells [reviewed in [32]]. The blue shift observed for the cAMP pathways in BSM cells of asthma patients, which indicates reduced activity, is in accord with reports showing a deficiency of the GPCR-cAMP-coupled signalling systems in BSM cells of asthma patients [33, 34].

Our unbiased, hypothesis-free high-throughput approach enabled us to uncover several additional molecular signalling pathways that have never been published in association with asthma. Here, the ILK pathway stands out with eleven up-regulated branches. Integrin-linked kinase (ILK) is a multi-domain protein kinase that binds to the cytoplasmic tail of beta-integrins and has been identified as an important mediator of signalling pathways that regulate the growth and differentiation state of airway smooth muscle. ILK protein overexpression in BSM cells results in smooth muscle-specific regulation of gene and protein expression [35]. Another signalling pathway of interest is the Aryl Hydrocarbon Receptor (AHR) pathway, which according to our results is characterized by five up-regulated branches. The AHR is a member of the bHLH (basic Helix-Loop-Helix)-PAS (PerARNT-Sim) family of transcriptional regulators, which has been described to be involved in allergic [36] and lung-inflammatory responses [37]. Up-regulation of estrogen pathway represents another intriguing observation as well as this pathway is elicited by estrogen receptors and linked –again—to the cAMP pathway to reduce intracellular Ca2+ levels, thus promoting bronchodilation [38]. Finally, we should mention that the nine-branched HIF-1α/Hypoxia pathways were up-regulated in BSM cells of asthma patients. These pathways are associated with and/or promote tissue remodelling processes, including extra-cellular matrix deposition, angiogenesis and cell proliferation. All these aforementioned pathways are unexpectedly up-regulated in BSM cells of asthma patients and might hence clarify their primed phenotype [1-5].

The hierarchical clustering analysis (Supplementary Figures S3 and S4) showed that the activity of 145 signalling pathways forms 7 clearly defined clusters, each characterized by concordant activation signatures of the enclosing pathways (Figure 3 and Supplementary Figure S2). Although congruent activation might be explained by the similarities in gene content between the cluster-forming pathways (e.g. AKT pathway terminal branches from cluster 1 or from cluster 2, or ILK pathway branches from cluster 3), most clusters represent the true functional coordination between the cluster members. Interestingly, the regulation of different functional clusters could be either congruent or opposed to each other (Figure 4). Unexpectedly, some clusters enclose pathways having rather opposite molecular functions. For example, Cluster 1 (Supplementary Table S6) includes two pathways leading to apoptosis: a branch of mitochondrial apoptosis pathway and an apoptotic branch of NGF pathway. They are compensated by the two anti-apoptotic branches of AKT pathway from the same cluster, one – directly inhibiting apoptosis, and another – promoting expression of cell survival genes. Moreover, Cluster 1 includes two additional pathways which attenuate cell growth and proliferation: a branch of GSK3 pathway leading to degradation of Beta-Catenin and a branch of SMAD pathway responsible for degradation of growth factor membrane receptors. These activities are, in turn, met by the cell growth and proliferation-promoting pathways from the same cluster: a branch of AKT that activates ERK signalling, and a branch of cAMP pathway that degrades cell cycle checkpoint regulators. Something similar may account for the other Clusters. For instance, in Cluster 2 a plethora of cell growth-, proliferation- and survival–promoting terminal branches of AKT pathway are juxtaposed to a cell cycle arrest-promoting pathway SMAD. Further in-depth analysis of all functional clusters may reveal previously unknown functional ensembles of tightly co-regulated signalling pathways.

In conclusion, our study is the first to explore the complete assortment of signalling pathways activated in primary human BSM cells of asthmatics relative to non-asthmatic subjects and provides a basis to understand the “primed” phenotype of asthmatic BSM cells. This exploration may be pivotal to development of novel therapeutic strategies that specifically address the pathways associated with pathophysiology of bronchomotor tone, airway hyper-responsiveness, as well as airway inflammation and airway remodelling.

## MATERIALS AND METHODS

### Tissue specimens and cell cultures

Lung tissue specimens were obtained from the Department of Internal Medicine, Pulmonology, University Hospital Basel, Basel, Switzerland with the approval of the local ethical committees and written consent of all patients. Primary BSM cultures were from non-asthmatic donors (selection criteria are listed in the Eurotransplant guidelines and include the absence of primary lung disease, such as asthma and chronic obstructive pulmonary disease (COPD), and ≤20 pack yr of smoking history). BSM cells were established as previously described [3] and grown in RPMI 1640 (Lonza, Basel, Switzerland) supplemented with 5% fetal calf serum (FCS), 8 mM L-glutamine, 20 mM hydroxyethyl piperazine ethane sulfonic acid and 1% modified Eagle's medium vitamin mix (Gibco, Paisley, UK). Neither antibiotics nor antimycotics were added at any time.

### RNA isolation

RNA isolation was performed with mirVana miRNA isolation kit (Ambion, Life Science, Zug, Switzerland) as described earlier [39]. For more details see Supplementary Material.

### CAGE sequencing and data analysis

Identification of primary BSM cells transcriptomes was performed by next-generation sequencing that allows a dynamic range of detection and measurement of relatively limited differences in expression between samples. CAGE library preparation was performed essentially as described [23]. For more details see Supplementary Material.

Raw sequencing data are available in NCBI Gene Expression Omnibus (GEO) database (http://www.ncbi.nlm.nih.gov/gds/) with Accession Number GSE63744.

### Differential expression analysis and pathway analysis

Raw reads were filtered by quality >30 score through FASTX toolkit [40] and then trimmed at 5′ and 3′ in order to remove index and adapter. Only the remaining reads were used for alignment with the human genome assembly (GRCh37) where we employed TopHat v2.0.14 [41]. CAGE tags were aligned to the human genome and the location of each tag respect to the nearest transcript annotated in Ensembl database [42] was calculated. The expression value of tags that aligned to multiple loci was distributed between these according to the weighting strategy of TopHat [41]. As promoters can vary in architecture, with some transcription units showing a strong preference for a particular base composition in its transcription initiation, and others using a broad collection of transcription start sites (TSSs) within a region of approximately 100 bases, TSSs have been clustered together when they mapped within 20 bases from each other [43]. Transcript expression values were normalized for each sample, resulting in values expressed in cluster tags per million (tpm). For more detailed description of methods used for differentially expression analysis see Supplementary Material.

### Source datasets

The signalling pathways knowledge base developed by SABiosciences (http://www.sabiosciences.com/pathwaycentral.php) was used to determine structures of intracellular pathways, which were used for OncoFinder, as described previously [15, 16].

### Functional annotation of gene expression data

For the functional annotation of the primary gene expression data, we applied our original algorithm termed OncoFinder [15, 16]. It enables calculation of the Pathway Activation Strength (PAS), a value which serves as a qualitative measure of pathway activation. Briefly, the enclosing algorithm utilizes the following formula to evaluate pathway activation:

$$\text{PA}S_{p}=\sum_{n}\text{AR}R_{\text{np}}\cdot \text{BTI}F_{n}\cdot \text{lg}(\text{CN}R_{n})$$

Here, the case-to-normal ratio (*CNRn*) is the ratio of expression levels for a gene *n* in the sample under investigation to the same average value for the control group of samples. The Boolean flag of *BTIF* (beyond tolerance interval flag) equals to one for genes with significantly altered expression, and to zero for non-significantly affected genes. The applied significance criteria were as follows: differential gene had to meet simultaneously the two conditions, first, gene expression level for the sample must lie outside the tolerance interval (p<0.05), and second, the value of *CNR* must differ from 1 considerably, thus being inferior of 0.66 or exceeding 1.5. The discrete value of *ARR* (activator/repressor role) reflects the functional role of a protein *n* in the pathway [15, 16].

### Statistical tests

The PAS values for each normal sample were obtained using the whole set of these normal samples as a reference. Distribution of PAS values was estimated, assuming its Gaussian behaviour. Then, for each pathway of each asthma sample, a probability that its PAS value comes from this estimated distribution was calculated. Additionally, p-values and FDRs for each pathway of the entire group of asthma samples were calculated using Wilcoxon rank-sum test and Benjamin & Hochberg method, respectively. Principal component analyses were performed using MADE4 package [44]. Hierarchical clustering heatmaps with Pearson distance and average linkage were generated using heatmap.2 function from “gplots” package [45]. Pearson tau correlation matrix was calculated in R 3.1.1 using a function of standard library “cor” with the default settings. Correlation diagram was built using a function “corrplot” from the package “corrplot” sorted with respect to hierarchical clustering. Similarities between the pathways according to the content of similar genes were calculated using Jaccard coefficient. The Jaccard coefficient measures similarity between finite sample sets, and is defined as the size of the intersection divided by the size of the union of the sample sets.

## SUPPLEMENTARY TABLES













## Abbreviations

BSM: bronchial smooth muscle
CAGE: cap analysis of gene expression
CTC: CAGE tag cluster
FCS: fetal calf serum
FEV1: Forced Expiratory Volume in 1 second
PAS: pathway activation strength
UTR: untranslated region.
